# Poster Session II - A309 CROHN’S DISEASE OF THE POUCH: A RETROSPECTIVE CASE SERIES FROM A CANADIAN TERTIARY CENTRE

**DOI:** 10.1093/jcag/gwaf042.309

**Published:** 2026-02-13

**Authors:** A Wadhwani, S Alobaid, R Mortuza, C Townsend, T Ponich, R Khanna, V Jairath, R Sedano

**Affiliations:** Medicine, Western University Schulich School of Medicine & Dentistry, London, ON, Canada; Medicine, Western University Schulich School of Medicine & Dentistry, London, ON, Canada; Medicine, Western University Schulich School of Medicine & Dentistry, London, ON, Canada; Medicine, Western University Schulich School of Medicine & Dentistry, London, ON, Canada; Medicine, Western University Schulich School of Medicine & Dentistry, London, ON, Canada; Medicine, Western University Schulich School of Medicine & Dentistry, London, ON, Canada; Medicine, Western University Schulich School of Medicine & Dentistry, London, ON, Canada; Medicine, Western University Schulich School of Medicine & Dentistry, London, ON, Canada

## Abstract

**Background:**

With an incidence of 5%-15%, Crohn’s Disease of the Pouch (CDP) is a rare, clinically challenging complication of ileal pouch–anal anastomosis (IPAA) in patients initially diagnosed with ulcerative colitis. Limited understanding of the mechanisms underlying CDP prevent the advancement of targeted therapies and standardized guidelines for diagnostic criteria and subsequent treatment approaches.

**Aims:**

Our study aimed to evaluate patients with documented CDP to identify pre-, intra-, and postoperative characteristics associated with disease control. Preoperative disease characteristics, intraoperative findings, and postoperative pharmacological therapy decisions were assessed in correlation to clinically active disease.

**Methods:**

A retrospective chart review was conducted on 26 patients diagnosed with CDP between 2005 and 2025 at London Health Sciences Center and St. Joseph’s Healthcare in London, Ontario. Demographic, preoperative, intraoperative, endoscopic, histologic, and pharmacologic data were collected. The primary outcome was clinical disease control status, defined as symptomatic remission with or without endoscopic remission. The data are presented as mean with standard deviation (SD) for normally distributed continuous variables. Categorical data is presented by absolute and relative frequencies (n and %). To account for associations between outcomes and covariates, we used chi square test.

**Results:**

Among 26 patients (mean age 51.5 ± 10.7 years; 53.8% male), 88.9% had preoperative pancolitis and underwent a staged IPAA. Clinical disease control was achieved in 69% of included patients and was associated with fewer surgical interventions (p = 0.026), fewer imaging abnormalities (p = 0.009), and distinct medication patterns (p = 0.001). Biologic therapy, predominantly infliximab, was more frequently used among well-controlled patients, whereas steroids and antibiotics were more commonly used in uncontrolled disease and in patients with pouch loss (p = 0.01). Pouch retention was achieved in 81.5% and was inversely associated with prior surgical complications (p = 0.047).

**Conclusions:**

Conclusion: Well-controlled CDP was associated with fewer postoperative surgical interventions and an improved response to biologic therapy (infliximab), demonstrating the importance of early and sustained biologic response in maintaining disease control and preventing complications. Larger, multicenter studies are needed to confirm these associations and refine postoperative treatment strategies, thereby contributing to the development of standardized CDP management guidelines. Our findings are limited in generalizability due to the small sample size.

**Funding Agencies:**

None

